# The incidence of acute myocardial infarction after elective spinal fusions or joint replacement surgery in the United States: a large-scale retrospective observational cohort study in 322,585 patients: Post-surgical myocardial infarction data

**DOI:** 10.1186/s13037-021-00305-6

**Published:** 2021-09-18

**Authors:** Patrick J. Arena, Jingping Mo, Qing Liu, Xiaofeng Zhou, Richard Gong, Charles Wentworth, Sundaresan Murugesan, Kui Huang

**Affiliations:** 1 Global Medical Epidemiology & Big Data Analysis, Pfizer Inc., 235 E 42nd St, New York, NY 10017 USA; 2 Safety Surveillance Research, Pfizer Inc., New York, NY USA; 3 Global Medical Epidemiology & Big Data Analysis, Pfizer Inc., Collegeville, PA USA; 4 Real World Evidence Center of Excellence, Pfizer Inc., New York, NY USA

**Keywords:** Myocardial infarction, Spinal fusion, Total hip arthroplasty, Total knee arthroplasty, Epidemiology, Electronic healthcare records

## Abstract

**Background:**

Acute myocardial infarction (AMI) is an uncommon but fatal complication among patients undergoing elective spinal fusion surgery (SF), total hip arthroplasty (THA), and total knee arthroplasty (TKA). Our objective was to estimate the incidence of AMI among adults undergoing elective SF, THA, and TKA in different post-operative risk windows and characterize high-risk sub-populations in the United States.

**Methods:**

A retrospective cohort study was conducted using data from a longitudinal electronic healthcare record (EHR) database from January 1, 2007 to June 30, 2018. ICD codes were used to identify SF, THA, TKA, AMI, and selected clinical characteristics. Incidence proportions (IPs) and 95% confidence intervals were estimated in the following risk windows: index hospitalization, ≤ 30, ≤ 90, ≤ 180, and ≤ 365 days post-operation.

**Results:**

A total of 67,533 SF patients, 87,572 THA patients, and 167,480 TKA patients were eligible for the study. The IP of AMI after SF, THA, and TKA ranged from 0.36, 0.28, and 0.25% during index hospitalization to 1.05, 0.93, and 0.85% ≤ 365 days post-operation, respectively. The IP of AMI was higher among patients who were older, male, with longer hospital stays, had a history of AMI, and had a history of diabetes.

**Conclusion:**

The IP of post-operative AMI was generally highest among the SF cohort compared to the THA and TKA cohorts. Additionally, potential high-risk populations were identified. Future studies in this area are warranted to confirm these findings via improved confounder control and to identify effect measure modifiers.

**Supplementary Information:**

The online version contains supplementary material available at 10.1186/s13037-021-00305-6.

## Background

Cardiovascular disease (CVD) is one of the top causes of morbidity and mortality globally [1]. Within the United States (US), the 2019 Heart Disease and Stroke Statistics report from the American Heart Association concluded that the prevalence of overall CVD (i.e., coronary heart disease, heart failure, stroke, and hypertension) in adults greater than 20 years of age was 48.0% (121.5 million Americans in 2016) [2]. Myocardial infarction (MI) is a key component to the burden of CVD [3]. Annually, over 800,000 people in the US experience an acute MI (AMI), of which 27% die [4].

Although AMI is an uncommon but fatal complication among elective surgical populations [5–7], the recognition of this adverse event is critical given the increased age of the population undergoing elective surgical procedures and the increased volume of surgeries in general. For example, recent research has shown that the frequency and utilization of spinal fusion (SF) have increased at a higher rate than other notable inpatient procedures (including laminectomy, percutaneous transluminal coronary angioplasty, and coronary artery bypass graft) over the period from 1998 to 2008 in the US; additionally, the authors found that the average age for SF increased over the study period [8]. Furthermore, an analysis of National Inpatient Sample (NIS) data from 2000 to 2014 determined that primary total hip arthroplasty (THA) is projected to grow 71% by 2030 and primary total knee arthroplasty (TKA) is projected to grow 85% by 2030 [9].

It is important to have background epidemiology data about post-operative AMI during various risk windows and among different sub-groups to contextualize safety data in clinical trials and the general population. This cohort study built on previous research [10], and it was designed to estimate the incidence of AMI among adults undergoing elective SF, THA, or TKA using a large electronic healthcare record (EHR) database in the US. The objectives of this study were to estimate the incidence of AMI among adults undergoing elective SF, THA, or TKA in different post-operative periods and to characterize high-risk sub-populations in the US.

## Methods

### Study design

A retrospective cohort study of adults undergoing elective SF, THA, or TKA using Optum EHR, a longitudinal US EHR database, was performed. Optum EHR partners directly with several multi-specialty medical groups, integrated delivery networks, and hospital chains throughout the US to extract their EHR data. By normalizing, validating, and aggregating the de-identified data, the database generates a longitudinal view of patient care and captures a comprehensive collection of demographic, clinical, operational, and financial information. As of June 2018, Optum EHR reported having data on approximately 94.4 million unique patients. Furthermore, about 40% of the patient population was aged 50 years or older, with approximately 15% of patients 65 years of age or older. About one quarter of the patients had at least 6 years of observation time within the database.

The study period was January 1, 2007 to June 30, 2018 with a length of follow-up equating to 365 days. The index date was defined as the first date after January 1, 2007 that an adult had undergone an elective SF, THA, or TKA during the study period. To incorporate a 183-day look-back window prior to the index surgery (for the purpose of obtaining medical history and excluding prevalent conditions as necessary), the earliest possible index surgical date was July 1, 2007. The latest index surgical date possible was June 30, 2017.

### Cohort formation

Eligible patients were 18 to 85 years of age at the time of their first elective SF, THA, or TKA in the database. Furthermore, patients had 183 days of continuous enrollment within the database prior to their first elective SF, THA, or TKA (i.e., the baseline period) as well as this index surgery being performed on the day of admission to the healthcare facility or the day after admission. Elective SF, THA, and TKA (i.e., the exposures) were identified using the International Classification of Diseases (ICD), Ninth Revision, Procedure Classification System (ICD-9-PCS) codes listed in Table 1.

**Table 1:** International Classification of Diseases (ICD), Ninth Revision, Procedure Classification System (ICD-9-PCS) codes used to define exposures

**Surgery Type**	**ICD-9-PCS Code**	**Code Description**
Spinal fusion (SF)	81.01	Atlas-axis spinal fusion
	81.02	Other cervical fusion of the anterior column, anterior technique
	81.03	Other cervical fusion of the posterior column, posterior technique
	81.04	Dorsal and dorsolumbar fusion of the anterior column, anterior technique
	81.05	Dorsal and dorsolumbar fusion of the posterior column, posterior technique
	81.06	Lumbar and lumbosacral fusion of the anterior column, anterior technique
	81.07	Lumbar and lumbosacral fusion of the posterior column, posterior technique
	81.08	Lumbar and lumbosacral fusion of the anterior column, posterior technique
	81.31	Refusion of atlas-axis spine
	81.32	Refusion of other cervical spine, anterior column, anterior technique
	81.33	Refusion of other cervical spine, posterior column, posterior technique
	81.34	Refusion of dorsal and dorsolumbar spine, anterior column, anterior technique
	81.35	Refusion of dorsal and dorsolumbar spine, posterior column, posterior technique
	81.36	Refusion of lumbar and lumbosacral spine, anterior column, anterior technique
	81.37	Refusion of lumbar and lumbosacral spine, posterior column, posterior technique
	81.38	Refusion of lumbar and lumbosacral spine, anterior column, posterior technique
	81.39	Refusion of spine, not elsewhere classified
	81.62	Fusion or refusion of 2–3 vertebrae
	81.63	Fusion or refusion of 4–8 vertebrae
	81.64	Fusion or refusion of 9 or more vertebrae
	84.51	Insertion of interbody spinal fusion device
Total hip arthroplasty (THA)	00.70	Revision of hip replacement, both acetabular and femoral components
	00.71	Revision of hip replacement, acetabular component
	00.72	Revision of hip replacement, femoral component
	00.73	Revision of hip replacement, acetabular liner and/or femoral head only
	00.85	Resurfacing hip, total, acetabulum and femoral head
	00.86	Resurfacing hip, partial, femoral head
	00.87	Resurfacing hip, partial, acetabulum
	81.51	Total hip replacement
	81.52	Partial hip replacement
	81.53	Revision of hip replacement, not otherwise specified
Total knee arthroplasty (TKA)	00.80	Revision of knee replacement, total (all components)
	00.81	Revision of knee replacement, tibial component
	00.82	Revision of knee replacement, femoral component
	00.83	Revision of knee replacement, patellar component
	00.84	Revision of total knee replacement, tibial insert (liner)
	81.54	Total knee replacement
	81.55	Revision of knee replacement, not otherwise specified

To select for a relatively healthy cohort that underwent inpatient elective surgeries, patients were excluded if they 1) underwent a major surgical procedure that occurred within 90 days prior to the index surgery; 2) had a surgical indication that was for an emergency procedure; 3) were pregnant; or 4) had cancer or end stage renal disease during the baseline period. AMI (i.e., the outcome) was defined based on the ICD, Ninth Revision, Clinical Modification (ICD-9-CM) codes listed in Table 2.

**Table 2:** International Classification of Diseases, Ninth Revision, Clinical Modification (ICD-9-CM) codes used to define acute myocardial infarction (AMI)

**ICD-9-CM Code**	**Code Description**
410.00	Acute myocardial infarction of anterolateral wall, episode of care unspecified
410.01	Acute myocardial infarction of anterolateral wall, initial episode of care
410.10	Acute myocardial infarction of other anterior wall, episode of care unspecified
410.11	Acute myocardial infarction of other anterior wall, initial episode of care
410.20	Acute myocardial infarction of inferolateral wall, episode of care unspecified
410.21	Acute myocardial infarction of inferolateral wall, initial episode of care
410.30	Acute myocardial infarction of inferoposterior wall, episode of care unspecified
410.31	Acute myocardial infarction of inferoposterior wall, initial episode of care
410.40	Acute myocardial infarction of other inferior wall, episode of care unspecified
410.41	Acute myocardial infarction of other inferior wall, initial episode of care
410.50	Acute myocardial infarction of other lateral wall, episode of care unspecified
410.51	Acute myocardial infarction of other lateral wall, initial episode of care
410.60	True posterior wall infarction, episode of care unspecified
410.61	True posterior wall infarction, initial episode of care
410.70	Subendocardial infarction, episode of care unspecified
410.71	Subendocardial infarction, initial episode of care
410.80	Acute myocardial infarction of other specified sites, episode of care unspecified
410.81	Acute myocardial infarction of other specified sites, initial episode of care
410.90	Acute myocardial infarction of unspecified site, episode of care unspecified
410.91	Acute myocardial infarction of unspecified site, initial episode of care

All ICD-9-PCS and ICD-9-CM codes were mapped to corresponding ICD, Tenth Revision (ICD-10) codes using General Equivalence Mapping techniques in order to account for the switch to ICD-10 coding in 2015.

### Data management and analysis

All analyses were descriptive and conducted in SAS (version 9.4, SAS Institute, Cary, NC, USA). Descriptive statistics were performed to characterize the cohort in terms of demographic and clinical characteristics at the baseline. Patients were followed from the cohort entry index date until the occurrence of AMI, death, loss to follow-up, or end of study period, whichever occurred first.

IPs were defined as the number of new cases of AMI during each specified post-surgical time interval divided by the total (AMI-free) population at the start of each time interval. Crude IPs were calculated overall, and in the following stratifications: age, sex, race, length of hospital stay, and selected clinical characteristics. IPs were also estimated in the following risk windows: index hospitalization (defined as the time interval from index surgery to discharge), ≤ 30 days (i.e., 0 to 30 days), ≤ 90 days (i.e., 0 to 90 days), ≤ 180 days (i.e., 0 to 180 days), and ≤ 365 days (i.e., 0 to 365 days) post-operation. For each post-operation period, incidence was calculated cumulatively; therefore, persons at risk and AMI events that were included in the preceding risk window were not excluded in the incidence calculation for the following risk window. All IPs were estimated with associated 95% confidence intervals (CIs), assuming a Poisson distribution.

Incidence rates were calculated as the number of new cases of AMI during each specified post-surgical time interval divided by the summed person-time of observation for the total (AMI-free) population at the start of each time interval. Although incidence rates are preferred over incidence proportions when there is long-term follow up (i.e., > 30 days), nearly all the literature identified in this area presented information in the form of incidence proportions; thus, only IPs are presented in the Results in order to facilitate better comparisons with the literature. However, incidence rate information is contained in the Additional file 1.

## Results

A total of 67,533 SF patients, 87,572 THA patients, and 167,480 TKA patients were eligible for the study. The median age at the index date was 59, 65, and 66 years for SF, THA, and TKA patients, respectively; there were more white patients (> 88.00%) across all surgical types, slightly more females undergoing THA (53.23%) and TKA (59.73%), and slightly more males undergoing SF (56.95%). Some of the most prevalent medical conditions included type 2 diabetes (13.91% for SF, 12.17% for THA, and 16.22% for TKA), cardiac dysrhythmias (9.12% for SF, 12.45% for THA, and 13.09% for TKA), chronic ischemic disease (8.86% for SF, 8.91% for THA, and 9.36% for TKA), hypothyroidism (7.06% for SF, 9.56% for THA, and 10.80% for TKA), and anemia (7.14% for SF, 15.14% for THA, and 12.84% for TKA). Within all three surgical cohorts, a history of AMI was rare (i.e., between 0.52 and 0.62% across cohorts). Dementia, deep vein thrombosis, and pulmonary embolism were also rare among all three surgical cohorts. The average length of hospital stay (LHS) (for the index hospitalization) was 3.41, 2.69, and 2.89 days for SF, THA, and TKA, respectively. Lastly, although less than 1.00% of SFs were revisional, 39.88% of THAs and 37.66% of TKAs were revisional surgeries. Table 3 shows the baseline demographics and clinical/surgical characteristics of the elective SF, THA, and TKA patient population.

**Table 3:** Baseline demographics, clinical characteristics, and surgical characteristics of the elective spinal fusion, total hip arthroplasty, and total knee arthroplasty cohorts

**Characteristic, N (%) except where specified**	**Spinal fusion**		**Total hip replacement**		**Total knee replacement**	
	**Number of patients**	**%**	**Number of patients**	**%**	**Number of patients**	**%**
Total	67,533		87,572		167,480	
**Demographic characteristics**						
Age at index date						
*Mean (SD)*	58.27 (13.37)		64.45 (11.00)		65.79 (9.48)	
*Median (Min, Max)*	59 (18,85)		66 (18,85)		66 (18,85)	
≥ 18–55	26,790	39.67	17,639	20.14	23,876	14.26
56–65	18,559	27.48	28,302	32.32	56,810	33.92
66–75	16,114	23.86	26,419	30.17	58,340	34.83
76–< 86	6,070	8.99	15,212	17.37	28,454	16.99
Sex						
Male	38,457	56.95	40,938	46.75	67,397	40.24
Female	29,053	43.02	46,611	53.23	100,028	59.73
Unknown	23	0.03	23	0.03	55	0.03
Race						
White	59,936	88.75	78,802	89.99	148,096	88.43
Black or African American	4,417	6.54	5,757	6.57	11,466	6.85
Asian	372	0.55	228	0.26	1,110	0.66
Other/Unknown	2,808	4.16	2,785	3.18	6,808	4.06
**Clinical characteristics in 183-day baseline**						
Acute myocardial infarction	418	0.62	490	0.56	869	0.52
Anemia	4,819	7.14	13,262	15.14	21,504	12.84
Angina	3,533	5.23	3,533	4.03	8,063	4.81
Asthma/wheezing/bronchospasm	5,284	7.82	6,382	7.29	13,425	8.02
Bronchitis/chronic obstructive pulmonary disease	3,849	5.70	4,722	5.39	8,212	4.90
Cardiac dysrhythmias	6,157	9.12	10,900	12.45	21,923	13.09
Chronic ischemic disease	5,984	8.86	7,807	8.91	15,669	9.36
Deep vein thrombosis	592	0.88	949	1.08	2,080	1.24
Dementia	218	0.32	380	0.43	516	0.31
Diabetes, type 1	480	0.71	331	0.38	774	0.46
Diabetes, type 2	9,392	13.91	10,659	12.17	27,164	16.22
Heart failure	1,458	2.16	2,400	2.74	4,657	2.78
Hyperthyroidism	191	0.28	381	0.44	731	0.44
Hypothyroidism	4,765	7.06	8,375	9.56	18,094	10.80
Intracranial hemorrhage	139	0.21	83	0.09	140	0.08
Liver disease and cirrhosis (excluding hepatitis, alcoholic liver disease)	1,023	1.51	1,206	1.38	2,347	1.40
Liver failure	50	0.07	67	0.08	111	0.07
Malignant neoplasms	0	0.00	0	0.00	0	0.00
Multiple sclerosis	186	0.28	172	0.20	282	0.17
Peripheral vascular disease	2,626	3.89	3,007	3.43	5,306	3.17
Psoriatic arthroplasty	72	0.11	87	0.10	197	0.12
Pulmonary embolism	247	0.37	402	0.46	1,015	0.61
Reactive arthritis	830	1.23	253	0.29	154	0.09
Renal failure	2,442	3.62	4,229	4.83	8,157	4.87
Respiratory failure	922	1.37	862	0.98	1,712	1.02
Spondyloarthritis, including ankylosing spondylitis	2,031	3.01	771	0.88	554	0.33
Stroke	529	0.78	580	0.66	1,094	0.65
**Surgical characteristics for index surgery**						
Allogenic blood transfusion during surgery	16	0.02	32	0.04	22	0.01
Existing permanently implanted device or prosthesis	1,839	2.72	2,149	2.45	3,470	2.07
Revisional surgery	538	0.80	34,924	39.88	63,065	37.66
Total length of hospital stay						
*Mean (SD)*	3.41 (3.93)		2.69 (2.42)		2.89 (2.21)	
*Median (Min, Max)*	3 (1,90)		2 (1,90)		3 (1,90)	
Use of implanted material during surgery	3,840	5.69	1,401	1.60	1,784	1.07

The IP of AMI following elective SF was 0.36% (95% CI: 0.31%, 0.41%) during initial hospitalization, 0.48% (95% CI: 0.43%, 0.54%) ≤ 30 days, 0.62% (95% CI: 0.56%, 0.68%) ≤ 90 days, 0.79% (95% CI: 0.72%, 0.86%) ≤ 180 days, and 1.05% (95% CI: 0.97%, 1.12%) ≤ 365 days post-operation. The IP of AMI following elective THA was 0.28% (95% CI: 0.24%, 0.31%) during initial hospitalization, 0.37% (95% CI: 0.33%, 0.41%) ≤ 30 days, 0.50% (95% CI: 0.45%, 0.55%) ≤ 90 days, 0.65% (95% CI: 0.60%, 0.70%) ≤ 180 days, and 0.93% (95% CI: 0.87%, 0.99%) ≤ 365 days post-operation. The IP of AMI following elective TKA was 0.25% (95% CI: 0.22%, 0.27%) during initial hospitalization, 0.34% (95% CI: 0.31%, 0.36%) ≤ 30 days, 0.43% (95% CI: 0.40%, 0.46%) ≤ 90 days, 0.56% (95% CI: 0.53%, 0.60%) ≤ 180 days, and 0.85% (95% CI: 0.80%, 0.89%) ≤ 365 days post-operation. Tables 4, 5, and 6 show the IP information for AMI among patients undergoing elective SF, THA, and TKA, respectively.

**Table 4:** Incidence proportion of acute myocardial infarction in patients undergoing elective spinal fusion during selected risk windows, crude and stratified by selected characteristics

	**During initial hospitalization**			**Up to 30 days post-operation**			**Up to 90 days post-operation**			**Up to 180 days post-operation**			**Up to 365 days post-operation**		
	**Number of patients at risk**	**Number of cases**	**Incidence proportion (95% confidence interval)**	**Number of patients at risk**	**Number of cases**	**Incidence proportion (95% confidence interval)**	**Number of patients at risk**	**Number of cases**	**Incidence proportion (95% confidence interval)**	**Number of patients at risk**	**Number of cases**	**Incidence proportion (95% confidence interval)**	**Number of patients at risk**	**Number of cases**	**Incidence proportion (95% confidence interval)**
**Crude**	67,020	241	0.36% (0.31%, 0.41%)	67,025	324	0.48% (0.43%, 0.54%)	67,028	417	0.62% (0.56%, 0.68%)	67,033	530	0.79% (0.72%, 0.86%)	67,034	702	1.05% (0.97%, 1.12%)
*Stratifications by:*															
**Age**															
≥ 18–55	26,702	27	0.10% (0.06%, 0.14%)	26,702	38	0.14% (0.10%, 0.19%)	26,703	52	0.20% (0.14%, 0.25%)	26,706	73	0.27% (0.21%, 0.34%)	26,707	108	0.40% (0.33%, 0.48%)
56–65	18,449	58	0.31% (0.23%, 0.40%)	18,450	80	0.43% (0.34%, 0.53%)	18,451	113	0.61% (0.50%, 0.73%)	18,452	148	0.80% (0.67%, 0.93%)	18,452	201	1.09% (0.94%, 1.24%)
66–75	15,924	96	0.60% (0.48%, 0.72%)	15,924	126	0.79% (0.65%, 0.93%)	15,925	154	0.97% (0.82%, 1.12%)	15,925	193	1.21% (1.04%, 1.38%)	15,925	247	1.55% (1.36%, 1.74%)
76–< 86	5,945	60	1.00% (0.76%, 1.26%)	5,949	80	1.35% (1.05%, 1.64%)	5,949	98	1.65% (1.32%, 1.97%)	5,950	116	1.95% (1.60%, 2.30%)	5,950	146	2.45% (2.06%, 2.85%)
**Sex**															
Male	38,149	171	0.45% (0.38%, 0.52%)	38,151	219	0.57% (0.50%, 0.65%)	38,153	285	0.75% (0.66%, 0.83%)	38,156	359	0.94% (0.84%, 1.04%)	38,157	482	1.26% (1.15%, 1.38%)
Female	28,848	70	0.24% (0.19%, 0.30%)	28,851	105	0.36% (0.30%, 0.43%)	28,852	132	0.46% (0.38%, 0.54%)	28,854	171	0.59% (0.50%, 0.68%)	28,854	219	0.76% (0.66%, 0.86%)
Unknown	23	0	0.00% (0.00%, 0.00%)	23	0	0.00% (0.00%, 0.00%)	23	0	0.00% (0.00%, 0.00%)	23	0	0.00% (0.00%, 0.00%)	23	1	4.35% (0.11%, 21.95%)
**Race**															
White	59,479	212	0.36% (0.31%, 0.40%)	59,482	288	0.48% (0.43%, 0.54%)	59,483	371	0.62% (0.56%, 0.69%)	59,487	478	0.80% (0.73%, 0.88%)	59,488	637	1.07% (0.99%, 1.15%)
Black or African American	4,399	18	0.41% (0.22%, 0.60%)	4,399	20	0.46% (0.26%, 0.65%)	4,401	27	0.61% (0.38%, 0.84%)	4,402	33	0.75% (0.50%, 1.01%)	4,402	43	0.98% (0.69%, 1.27%)
Asian	356	4	1.11% (0.31%, 2.85%)	356	5	1.41% (0.46%, 3.25%)	356	5	1.40% (0.46%, 3.25%)	356	5	1.41% (0.46%, 3.25%)	356	5	1.41% (0.46%, 3.25%)
Other/Unknown	2,786	7	0.25% (0.07%, 0.44%)	2,788	11	0.50% (0.24%, 0.77%)	2,788	14	0.50% (0.27%, 0.77%)	2,788	14	0.50% (0.24%, 0.77%)	2,788	17	0.61% (0.32%, 0.90%)
**Type 1 Diabetes during baseline**															
Yes	480	6	1.25% (0.26%, 2.24%)	480	7	1.46% (0.39%, 2.53%)	480	9	1.88% (0.66%, 3.09%)	480	13	2.71% (1.26%, 4.16%)	480	18	3.75% (2.05%, 5.45%)
No	66,540	235	0.35% (0.31%, 0.40%)	66,545	317	0.48% (0.42%, 0.53%)	66,548	408	0.61% (0.55%, 0.67%)	66,553	517	0.78% (0.71%, 0.84%)	66,554	684	1.03% (0.95%, 1.11%)
**Type 2 Diabetes during baseline**															
Yes	9,204	65	0.71% (0.54%, 0.88%)	9,209	96	1.04% (0.84%, 1.25%)	9,210	127	1.38% (1.14%, 1.62%)	9,210	162	1.76% (1.49%, 2.03%)	9,210	213	2.31% (2.00%, 2.62%)
No	57,816	176	0.30% (0.26%, 0.35%)	57,816	228	0.39% (0.34%, 0.45%)	57,818	290	0.50% (0.44%, 0.56%)	57,823	368	0.64% (0.57%, 0.70%)	57,824	489	0.85% (0.77%, 0.92%)
**Revisional surgery on the same day as index surgery**															
Yes	534	3	0.56% (0.12%, 1.63%)	534	4	0.75% (0.21%, 1.91%)	534	4	0.75% (0.21%, 1.91%)	534	4	0.75% (0.21%, 1.91%)	534	6	1.12% (0.23%, 2.02%)
No	66,486	238	0.36% (0.31%, 0.40%)	66,491	320	0.48% (0.43%, 0.54%)	66,494	413	0.62% (0.56%, 0.68%)	66,499	526	0.79% (0.72%, 0.86%)	66,500	696	1.05% (0.97%, 1.12%)
**Total length of hospital stay**															
1–5 days	59,512	108	0.18% (0.15%, 0.22%)	59,516	174	0.29% (0.25%, 0.34%)	59,518	244	0.41% (0.36%, 0.46%)	59,522	341	0.57% (0.51%, 0.63%)	59,523	488	0.82% (0.75%, 0.89%)
6–10 days	5,715	67	1.17% (0.90%, 1.45%)	5,716	82	1.43% (1.13%, 1.74%)	5,717	196	1.68% (1.35%, 2.01%)	5,717	110	1.92% (1.57%, 2.28%)	5,717	124	2.17% (1.79%, 2.55%)
> 10 days	1,793	66	3.68% (2.81%, 4.55%)	1,793	68	3.79% (2.91%, 4.68%)	1,793	77	4.29% (3.36%, 5.23%)	1,794	79	4.40% (3.45%, 5.35%)	1,794	90	5.02% (4.00%, 6.03%)
**Use of implanted material during surgery on the same day as index surgery**															
Yes	3,799	13	0.34% (0.16%, 0.53%)	3,799	19	0.50% (0.28%, 0.73%)	3,799	22	0.58% (0.34%, 0.82%)	3,799	26	0.68% (0.42%, 0.95%)	3,799	34	0.90% (0.60%, 1.20%)
No	63,221	228	0.36% (0.31%, 0.41%)	63,226	305	0.48% (0.43%, 0.54%)	63,229	395	0.62% (0.57%, 0.69%)	63,234	504	0.80% (0.73%, 0.87%)	63,235	668	1.06% (0.98%, 1.14%)
**Medical history of acute myocardial infarction**															
Yes	415	53	12.77% (9.56%, 15.98%)	416	66	15.87% (12.35%, 19.38%)	416	81	19.47% (15.67%, 23.28%)	416	93	22.36% (18.35%, 26.36%)	416	115	27.64% (23.25%, 31.94%)
No	66,605	188	0.28% (0.24%, 0.32%)	66,609	258	0.39% (0.34%, 0.44%)	66,612	336	0.50% (0.45%, 0.56%)	66,617	437	0.66% (0.60%, 0.72%)	66,618	587	0.88% (0.81%, 0.95%)

**Table 5:** Incidence proportion of acute myocardial infarction in patients undergoing elective total hip arthroplasty during selected risk windows, crude and stratified by selected characteristics

	**During initial hospitalization**			**Up to 30 days post-operation**			**Up to 90 days post-operation**			**Up to 180 days post-operation**			**Up to 365 days post-operation**		
	**Number of patients at risk**	**Number of cases**	**Incidence proportion (95% confidence interval)**	**Number of patients at risk**	**Number of cases**	**Incidence proportion (95% confidence interval)**	**Number of patients at risk**	**Number of cases**	**Incidence proportion (95% confidence interval)**	**Number of patients at risk**	**Number of cases**	**Incidence proportion (95% confidence interval)**	**Number of patients at risk**	**Number of cases**	**Incidence proportion (95% confidence interval)**
**Crude**	86,529	239	0.28% (0.24%, 0.31%)	86,530	318	0.37% (0.33%, 0.41%)	86,530	432	0.50% (0.45%, 0.55%)	86,532	561	0.65% (0.59%, 0.70%)	86,535	805	0.93% (0.87%, 0.99%)
*Stratifications by:*															
**Age**															
≥ 18–55	17,539	12	0.07% (0.03%, 0.11%)	17,539	18	0.10% (0.06%, 0.15%)	17,539	33	0.19% (0.12%, 0.25%)	17,539	45	0.26% (0.18%, 0.33%)	17,541	70	0.40% (0.31%, 0.49%)
56–65	28,025	42	0.15% (0.10%, 0.20%)	28,026	58	0.21% (0.15%, 0.26%)	28,026	86	0.31% (0.24%, 0.37%)	28,027	120	0.43% (0.35%, 0.50%)	28,027	183	0.65% (0.56%, 0.75%)
66–75	26,043	84	0.32% (0.25%, 0.39%)	26,043	105	0.40% (0.33%, 0.48%)	26,043	136	0.52% (0.43%, 0.62%)	26,044	186	0.71% (0.61%, 0.82%)	26,044	257	0.99% (0.87%, 1.11%)
76–< 86	14,922	101	0.68% (0.55%, 0.81%)	14,922	137	0.92% (0.77%, 1.07%)	14,922	177	1.19% (1.01%, 1.36%)	14,922	210	1.41% (1.22%, 1.60%)	14,923	295	1.98% (1.75%, 2.20%)
**Sex**															
Male	40,445	123	0.30% (0.25%, 0.36%)	40,446	169	0.42% (0.36%, 0.48%)	40,446	238	0.59% (0.51%, 0.66%)	40,447	318	0.79% (0.70%, 0.87%)	40,450	471	1.16% (1.06%, 1.27%)
Female	46,062	115	0.25% (0.21%, 0.30%)	46,062	148	0.32% (0.27%, 0.37%)	46,062	193	0.42% (0.36%, 0.48%)	46,063	242	0.53% (0.46%, 0.59%)	46,063	333	0.72% (0.65%, 0.80%)
Unknown	22	1	4.55% (0.12%, 22.84%)	22	1	4.55% (0.12%, 22.84%)	22	1	4.55% (0.12%, 22.84%)	22	1	4.55% (0.12%, 22.84%)	22	1	4.55% (0.12%, 22.84%)
**Race**															
White	77,834	221	0.28% (0.25%, 0.32%)	77,835	293	0.38% (0.33%, 0.42%)	77,835	397	0.51% (0.46%, 0.56%)	77,836	509	0.65% (0.60%, 0.71%)	77,838	731	0.94% (0.87%, 1.01%)
Black or African American	5,729	10	0.17% (0.07%, 0.28%)	5,729	15	0.26% (0.13%, 0.39%)	5,729	23	0.40% (0.24%, 0.57%)	5,729	35	0.61% (0.41%, 0.81%)	5,729	48	0.84% (0.60%, 1.07%)
Asian	209	1	0.48% (0.01%, 2.64%)	209	1	0.48% (0.01%, 2.64%)	209	1	0.48% (0.01%, 2.64%)	210	2	0.95% (0.12%, 3.40%)	210	2	0.95% (0.12%, 3.40%)
Other/Unknown	2,757	7	0.25% (0.07%, 0.44%)	2,757	9	0.33% (0.11%, 0.54%)	2,757	11	0.40% (0.16%, 0.63%)	2,757	15	0.54% (0.27%, 0.82%)	2,758	24	0.87% (0.52%, 1.22%)
**Type 1 Diabetes during baseline**															
Yes	337	8	2.37% (0.75%, 4.00%)	337	9	2.67% (0.95%, 4.39%)	337	10	2.97% (1.16%, 4.78%)	337	11	3.26% (1.37%, 5.16%)	337	13	3.86% (1.80%, 5.91%)
No	86,192	231	0.27% (0.23%, 0.30%)	86,193	309	0.36% (0.32%, 0.40%)	86,193	422	0.49% (0.44%, 0.54%)	86,195	550	0.64% (0.58%, 0.69%)	86,198	792	0.92% (0.86%, 0.98%)
**Type 2 Diabetes during baseline**															
Yes	10,364	57	0.55% (0.41%, 0.69%)	10,365	80	0.77% (0.60%, 0.94%)	10,365	101	0.97% (0.79%, 1.16%)	10,366	129	1.24% (1.03%, 1.46%)	10,367	200	1.93% (1.66%, 2.19%)
No	76,165	182	0.24% (0.20%, 0.27%)	76,165	238	0.31% (0.27%, 0.35%)	76,165	331	0.43% (0.39%, 0.48%)	76,166	432	0.57% (0.51%, 0.62%)	76,168	605	0.79% (0.73%, 0.86%)
**Revisional surgery on the same day as index surgery**															
Yes	34,555	72	0.21% (0.16%, 0.26%)	34,555	105	0.30% (0.25%, 0.36%)	34,555	148	0.43% (0.36%, 0.50%)	34,555	204	0.59% (0.51%, 0.67%)	34,555	276	0.80% (0.70%, 0.89%)
No	51,974	167	0.32% (0.27%, 0.37%)	51,975	213	0.41% (0.35%, 0.46%)	51,975	284	0.55% (0.48%, 0.61%)	51,977	357	0.69% (0.62%, 0.76%)	51,980	529	1.02% (0.93%, 1.10%)
**Total length of hospital stay**															
1–5 days	83,029	150	0.18% (0.15%, 0.21%)	83,030	225	0.27% (0.24%, 0.31%)	83,030	327	0.39% (0.35%, 0.44%)	83,032	449	0.54% (0.49%, 0.59%)	83,035	683	0.82% (0.76%, 0.88%)
6–10 days	2,425	58	2.39% (1.78%, 3.00%)	2,425	62	2.56% (1.93%, 3.18%)	2,425	70	2.89% (2.22%, 3.55%)	2,425	75	3.09% (2.40%, 3.78%)	2,425	84	3.46% (2.74%, 4.19%)
> 10 days	1,075	31	2.88% (1.88%, 3.88%)	1,075	31	2.88% (1.88%, 3.88%)	1,075	35	3.26% (2.19%, 4.32%)	1,075	37	3.44% (2.35%, 4.53%)	1,075	38	3.53% (2.43%, 4.64%)
**Use of implanted material during surgery on the same day as index surgery**															
Yes	1,383	19	1.37% (0.76%, 1.99%)	1,383	23	1.66% (0.99%, 2.34%)	1,383	27	1.95% (1.22%, 2.68%)	1,383	30	2.17% (1.40%, 2.94%)	1,384	37	2.67% (1.82%, 3.52%)
No	85,146	220	0.26% (0.22%, 0.29%)	85,147	295	0.35% (0.31%, 0.39%)	85,147	405	0.48% (0.43%, 0.52%)	85,149	531	0.62% (0.57%, 0.68%)	85,151	768	0.90% (0.84%, 0.97%)
**Medical history of acute myocardial infarction**															
Yes	485	64	13.20% (10.18%, 16.21%)	485	78	16.08% (12.81%, 19.35%)	485	95	19.59% (16.06%, 23.12%)	485	115	23.71% (19.93%, 27.50%)	485	145	29.90% (25.82%, 33.97%)
No	86,044	175	0.20% (0.17%, 0.23%)	86,045	240	0.28% (0.24%, 0.31%)	86,045	337	0.39% (0.35%, 0.43%)	86,047	446	0.52% (0.47%, 0.57%)	86,050	660	0.77% (0.71%, 0.83%)

**Table 6:** Incidence proportion of acute myocardial infarction in patients undergoing elective total knee arthroplasty during selected risk windows, crude and stratified by selected characteristics

	**During initial hospitalization**			**Up to 30 days post-operation**			**Up to 90 days post-operation**			**Up to 180 days post-operation**			**Up to 365 days post-operation**		
	**Number of patients at risk**	**Number of cases**	**Incidence proportion (95% confidence interval)**	**Number of patients at risk**	**Number of cases**	**Incidence proportion (95% confidence interval)**	**Number of patients at risk**	**Number of cases**	**Incidence proportion (95% confidence interval)**	**Number of patients at risk**	**Number of cases**	**Incidence proportion (95% confidence interval)**	**Number of patients at risk**	**Number of cases**	**Incidence proportion (95% confidence interval)**
**Crude**	165,863	406	0.24% (0.22%, 0.27%)	165,866	557	0.34% (0.31%, 0.36%)	165,866	717	0.43% (0.40%, 0.46%)	165,871	931	0.56% (0.53%, 0.60%)	165,879	1,407	0.85% (0.80%, 0.89%)
*Stratifications by:*															
**Age**															
≥ 18–55	23,770	16	0.07% (0.03%, 0.10%)	23,770	30	0.13% (0.08%, 0.17%)	23,770	40	0.17% (0.12%, 0.22%)	23,770	56	0.24% (0.17%, 0.30%)	23,770	88	0.37% (0.29%, 0.45%)
56–65	56,427	89	0.16% (0.13%, 0.19%)	56,427	132	0.23% (0.19%, 0.27%)	56,427	176	0.31% (0.27%, 0.36%)	56,427	229	0.41% (0.35%, 0.46%)	56,429	348	0.62% (0.55%, 0.68%)
66–75	57,710	155	0.27% (0.23%, 0.31%)	57,711	204	0.35% (0.31%, 0.40%)	57,711	259	0.45% (0.39%, 0.50%)	57,713	347	0.60% (0.54%, 0.66%)	57,716	522	0.90% (0.83%, 0.98%)
76–< 86	27,956	146	0.52% (0.44%, 0.61%)	27,958	191	0.68% (0.59%, 0.78%)	27,958	242	0.87% (0.76%, 0.97%)	27,961	299	1.07% (0.95%, 1.19%)	27,964	449	1.61% (1.46%, 1.75%)
**Sex**															
Male	66,721	230	0.34% (0.30%, 0.39%)	66,722	326	0.49% (0.44%, 0.54%)	66,722	422	0.63% (0.57%, 0.69%)	66,723	540	0.81% (0.74%, 0.88%)	66,727	825	1.24% (1.15%, 1.32%)
Female	99,087	176	0.18% (0.15%, 0.20%)	99,089	231	0.23% (0.20%, 0.26%)	99,089	295	0.30% (0.26%, 0.33%)	99,093	391	0.39% (0.36%, 0.43%)	99,097	582	0.59% (0.54%, 0.64%)
Unknown	55	0	0.00% (0.00%, 0.00%)	55	0	0.00% (0.00%, 0.00%)	55	0	0.00% (0.00%, 0.00%)	55	0	0.00% (0.00%, 0.00%)	55	0	0.00% (0.00%, 0.00%)
**Race**															
White	146,645	354	0.24% (0.22%, 0.27%)	146,647	488	0.33% (0.30%, 0.36%)	146,647	631	0.43% (0.40%, 0.46%)	146,651	826	0.56% (0.52%, 0.60%)	146,658	1,259	0.86% (0.81%, 0.91%)
Black or African American	11,417	32	0.28% (0.18%, 0.38%)	11,418	42	0.37% (0.26%, 0.48%)	11,418	51	0.45% (0.32%, 0.57%)	11,419	62	0.54% (0.41%, 0.68%)	11,419	94	0.82% (0.66%, 0.99%)
Asian	1,073	3	0.28% (0.06%, 0.81%)	1,073	4	0.37% (0.10%, 0.95%)	1,073	4	0.37% (0.10%, 0.95%)	1,073	4	0.37% (0.10%, 0.95%)	1,073	5	0.47% (0.15%, 1.08%)
Other/Unknown	6,728	17	0.25% (0.13%, 0.37%)	6,728	23	0.34% (0.20%, 0.48%)	6,728	31	0.46% (0.30%, 0.62%)	6,728	39	0.58% (0.40%, 0.76%)	6,729	49	0.73% (0.53%, 0.93%)
**Type 1 Diabetes during baseline**															
Yes	754	4	0.53% (0.14%, 1.35%)	754	9	1.19% (0.42%, 1.97%)	754	10	1.33% (0.65%, 2.14%)	754	11	1.46% (0.60%, 2.31%)	754	15	1.99% (0.99%, 2.99%)
No	165,109	402	0.24% (0.22%, 0.27%)	165,112	548	0.33% (0.30%, 0.36%)	165,112	707	0.43% (0.40%, 0.46%)	165,117	920	0.56% (0.52%, 0.59%)	165,125	1,392	0.84% (0.80%, 0.89%)
**Type 2 Diabetes during baseline**															
Yes	26,598	108	0.41% (0.33%, 0.48%)	26,599	158	0.59% (0.50%, 0.69%)	26,599	201	0.76% (0.65%, 0.86%)	26,601	264	0.99% (0.87%, 1.11%)	26,602	372	1.40% (1.26%, 1.54%)
No	139,265	298	0.21% (0.19%, 0.24%)	139,267	399	0.29% (0.26%, 0.31%)	139,267	516	0.37% (0.34%, 0.40%)	139,270	667	0.48% (0.44%, 0.52%)	139,277	1,035	0.74% (0.70%, 0.79%)
**Revisional surgery on the same day as index surgery**															
Yes	62,541	142	0.23% (0.19%, 0.26%)	62,543	189	0.30% (0.26%, 0.35%)	62,543	242	0.39% (0.34%, 0.44%)	62,543	315	0.50% (0.45%, 0.56%)	62,545	453	0.72% (0.66%, 0.79%)
No	103,322	264	0.26% (0.23%, 0.29%)	103,323	368	0.36% (0.32%, 0.39%)	103,323	475	0.46% (0.42%, 0.50%)	103,328	616	0.60% (0.55%, 0.64%)	103,334	954	0.92% (0.86%, 0.98%)
**Total length of hospital stay**															
1–5 days	158,267	238	0.15% (0.13%, 0.17%)	158,270	379	0.24% (0.22%, 0.26%)	158,270	529	0.33% (0.30%, 0.36%)	158,273	730	0.46% (0.43%, 0.49%)	158,281	1,164	0.74% (0.69%, 0.78%)
6–10 days	5,458	100	1.83% (1.48%, 2.19%)	5,458	109	2.00% (1.63%, 2.37%)	5,458	113	2.07% (1.69%, 2.45%)	5,458	120	2.20% (1.81%, 2.59%)	5,458	150	2.75% (2.31%, 3.18%)
> 10 days	2,138	68	3.18% (2.44%, 3.92%)	2,138	69	3.23% (2.48%, 3.98%)	2,138	75	3.51% (2.73%, 4.29%)	2,140	81	3.79% (2.98%, 4.59%)	2,140	93	3.48% (3.48%, 5.21%)
**Use of implanted material during surgery on the same day as index surgery**															
Yes	1,768	1	0.06% (0.00%, 0.31%)	1,768	4	0.23% (0.06%, 0.58%)	1,768	6	0.34% (0.07%, 0.61%)	1,768	10	0.57% (0.22%, 0.92%)	1,768	15	0.85% (0.42%, 1.28%)
No	164,095	405	0.25% (0.22%, 0.27%)	164,098	553	0.34% (0.31%, 0.37%)	164,098	711	0.43% (0.40%, 0.47%)	164,103	921	0.56% (0.53%, 0.60%)	164,111	1,392	0.85% (0.80%, 0.89%)
**Medical history of acute myocardial infarction**															
Yes	864	93	10.76% (8.70%, 12.83%)	864	101	11.69% (9.55%, 13.83%)	864	119	13.77% (11.48%, 16.07%)	864	153	17.71% (15.16%, 20.25%)	864	185	21.41% (18.68%, 24.15%)
No	164,999	313	0.19% (0.17%, 0.21%)	165,002	456	0.28% (0.25%, 0.30%)	165,002	598	0.36% (0.33%, 0.39%)	165,007	778	0.47% (0.44%, 0.51%)	165,015	1,222	0.74% (0.70%, 0.78%)

Stratified analyses revealed that the IP of AMI was higher among patients who were older, male, had a history of AMI, and had a history of diabetes across the three types of surgery at each risk window. For example, the IP of post-operative AMI among elective SF patients during index hospitalization was 0.10% (95% CI: 0.06%, 0.14%) among those aged ≥ 18 to 55 years, 0.31% (95% CI: 0.23%, 0.40%) among those aged 56 to 65 years, 0.60% (95% CI: 0.48%, 0.72%) among those aged 66 to 75 years, and 1.01% (95% CI: 0.76%, 1.26%) among those aged 76 to < 86 years. Moreover, the IP of post-operative AMI among elective THA patients ≤ 365 days post-operation was 1.16% (95% CI: 1.06%, 1.27%) for men and 0.72% (95% CI: 0.65%, 0.80%) for women. Additionally, the IP of AMI was higher among patients who had greater LHS across the three types of surgery at each risk window; however, it should be noted that these longer hospital stays could be a result of AMI instead of a reverse relationship (i.e., where AMI is the consequence of a longer hospital stay).

The IP of AMI was slightly higher among black patients for TKA in most risk windows and for SF up to 90 days post-operation compared to white patients, while the IP of AMI was higher among white patients for THA at each risk window. For instance, the IP of post-operative AMI among elective TKA patients ≤ 30 days post-operation was 0.37% (95% CI: 0.26%, 0.48%) for black patients and 0.33% (95% CI: 0.30%, 0.36%) for white patients. Generally, SF patients with same day revisional surgery had higher IPs of AMI across risk windows compared to patients who did not have same day revisional surgery; however, the opposite association was observed for THA and TKA patients (i.e., the IP of AMI was lower for those with same day revisional surgery compared to those who did not have same day revisional surgery across risk windows).

Detailed incidence rate information can be found in the Additional file 1 (i.e., Table S1, Table S2, and Table S3). The incidence rate of AMI per 1000 person-years (PYs) following elective SF was 18.12 (95% CI: 16.64, 19.73) ≤ 180 days post-operation and 12.82 (95% CI: 11.90, 13.80) ≤ 365 days post-operation. The incidence rate of AMI per 1000 PYs following elective THA 15.03 (95% CI: 13.83, 16.32) ≤ 180 days post-operation and 11.45 (95% CI: 10.69, 12.27) ≤ 365 days post-operation. The incidence rate of AMI per 1000 PYs following elective TKA was 12.77 (95% CI: 11.97, 13.61) ≤ 180 days post-operation and 10.17 (95% CI: 9.66, 10.72) ≤ 365 days post-operation. It should be highlighted that the incidence rate of AMI following elective surgery decreased consistently from index hospitalization to ≤ 365 days post-operation across all three surgical cohorts.

## Discussion

### Summary

This study identified 67,533 SF patients, 87,572 THA patients, and 167,480 TKA patients who underwent elective surgeries within the Optum EHR database. The IP of AMI following these elective procedures was generally highest among the SF cohort compared to the THA and TKA cohorts. When stratified by relevant demographic and clinical characteristics, we found that the IP of post-operative AMI was higher among patients who were older, male, with longer hospital stays, had a history of AMI, and had a history of diabetes.

### Spinal fusion cohort

Adogwa et al. performed a retrospective database study using the American College of Surgeons (ACS) National Surgical Quality Improvement Program (NSQIP) database from January 2008 through December 2014 and analyzed 23,102 patients undergoing lumbar decompression and fusion procedures; the authors found that patients with extended LHS had a higher incidence of post-operative MI: 0.16% for those with normal LHS and 1.29% for those with extended LHS [11]. Similarly, another investigation by Adogwa et al. of adult patients aged 65 and older in the ACS NSQIP database who underwent certain SF procedures (*n* = 4730) from 2008 through 2014 also found that patients with extended LHS had a higher incidence of post-operative MI: 0.06% for those with normal LHS and 1.18% for those with extended LHS [12]. In both studies, the authors stressed that much of the variation in LHS after spinal surgery was most likely due to differences in practice style or surgeon preference (as opposed to in-hospital complications or baseline comorbidities). In the same vein, Shen et al. conducted a retrospective cohort analysis of 254,640 patients undergoing elective SF in the Nationwide Inpatient Sample (NIS) from 2001 to 2005 and found that the IP of cardiovascular complications during hospitalization increased with age: 0.52% for those aged 20 to 24 years, 0.52% for those aged 35 to 49 years, 1.06% for those aged 50 to 64 years, 2.32% for those aged 64 to 74 years, and 3.20% for those aged 75 years or more [13]; although we used different age categorizations, we too found a similar relationship between age and the incidence of post-operative AMI.

Moreover, Chung et al. performed a retrospective cohort study of 15,618 patients undergoing elective SF in the ACS NSQIP between 2006 and 2013 and concluded that patients with metabolic syndrome had increased incidence of post-operative MI (0.5%) compared to those without metabolic syndrome (0.3%) [6]. Likewise, Memstoudis et al. also investigated the relation between post-operative MI and metabolic syndrome, but these authors used the NIS database from 1998 to 2008 and patients undergoing primary posterior lumbar fusion; nonetheless, Metmstoudis et al. also found that patients with metabolic syndrome had increased incidence of post-operative MI (0.6%) compared to those without metabolic syndrome (0.3%) [14]. Although we did not look at this condition explicitly, metabolic syndrome is a composite condition that includes diabetes; we found that patients with type 2 diabetes had increased incidence of post-operative AMI compared to those without type 2 diabetes, so our finding is in line with the Chung et al. and Memstoudis et al. studies. Lastly, Basques et al. performed a retrospective cohort study of the ACS NSQIP database from 2005 to 2013 and examined post-operative MI among patients that underwent primary and revisional posterior lumbar fusion; the authors found the 30-day incidence of MI was similar among patients who underwent primary posterior lumbar fusion (0.3%) compared to patients who underwent revision posterior lumbar fusion (0.2%) [15]. This finding somewhat aligns with the association that we found: the 30-day incidence of AMI was 0.75% among patients who underwent revisional SF and 0.48% among patients who did not undergo revisional SF. However, less than 600 patients underwent revisional SF in our cohort; therefore, the IP estimate may not be stable.

### Total hip arthroplasty and total knee arthroplasty cohorts

Kreder et al. evaluated 26,320 patients who underwent primary THA and TKA by performing a retrospective cohort study of administrative data in Ontario, Canada; the authors reported that the incidence of AMI increased with age among both THA patients (0.47% for those aged 65 to 79 years and 1.28% for those aged 80 years or greater) and TKA patients (0.45% for those aged 65 to 79 years and 1.09% for those aged 80 years or greater) [16]. Likewise, Koenig et al. performed a retrospective review of 306 patients who underwent revision THA; the authors found that the 90-day incidence of post-operative MI was 0.0% for those aged less than 65 years and 1.6% for those aged 65 to 79 years [17]. As was the case with SF cohort, we also found that the incidence of post-operative AMI increased with age in both the THA and TKA cohorts.

Moreover, Blum et al. conducted a retrospective cohort study of 17,385 patients that underwent primary TKA within the Pennsylvania Health Care Cost Containment Council data from 2001 to 2007; the authors found the 30-day incidence of post-operative MI was 0.5% for white patients and 0.2% for black patients [7]; their finding runs slightly counter to our own as we found that 30-day IP of post-operative AMI was 0.33% for white patients and 0.37% for black patients. However, it should be noted that these authors used only one ICD-9 code to define TKA (81.54), and that their analysis focused on one state as opposed to the entire US (which was the case in our study); nonetheless, we used virtually the same MI definitions [7]. With regards to sex-specific differences, Basques et al. performed a retrospective cohort study of elective THA and TKA patients using the NIS from 2002 to 2011 and concluded that the odds of MI among males were 1.6 times the odds among females [18]; we found a similar relationship in that the men in our study generally had higher incidences of AMI compared to women across all risk windows for both THA and TKA.

Furthermore, Pulido et al. performed an institutional review of their prospective database of patients undergoing elective joint arthroplasty and identified 15,383 patients who had THA or TKA; the authors found that the incidence of in-hospital MI was higher among revisional surgery than among primary surgery patients for both THA (0.35 vs. 0.16%) and TKA (0.62 and 0.33%) [19]. We found an opposite association for both TKA (0.23% for revisional surgery and 0.26% for non-revisional surgery) and THA (0.21% for revisional surgery and 0.32% for non-revisional surgery) during index hospitalization. However, it should be noted that their analysis comprised of one institution (as opposed to our hospital network-based analysis). Still, Khatod et al. performed a retrospective review of 17,080 primary and revisional TKAs at Southern California Kaiser Permanente between 1995 and 2004 and found that the post-operative incidences of MI were approximately the same for revisional and primary TKA [20], which is roughly in accordance with what was found for our TKA population. Nonetheless, Mohamed et al. conducted a retrospective cohort study of Medicare claims data in the year 2000 to identify patients undergoing primary and revision TKA and determined that the 90-day incidence of post-operative MI was 0.8% for primary TKA and 1.0% for revisional TKA [21]. We again see that our 90-day incidence finding (0.39% for revisional TKA compared to 0.43% for primary TKA) is not in line with the literature. Although Mohamed et al. also used a national database, they used both ICD-9 and Current Procedural Terminology (CPT) codes to identify TKA and only looked at data for the year 2000 (as opposed to the eleven-year period analyzed here) [21].

### Recurrent AMI

Although no studies looked at the incidence of post-operative AMI among patients with a history of AMI, it is well-established that patients who survive an AMI episode have an increased risk of a future AMI [22]. Thus, our analysis (among those with a history of AMI) agree with such a relationship as we found that patients with a history of AMI had a drastically higher incidence than those without such a history; for example, the IP of post-operative AMI during index hospitalization among the TKA cohort was 10.76% for those with a medical history of AMI compared to 0.19% for those without such a history.

### Database considerations

It is noteworthy that our study used Optum EHR database while most studies in the literature used NSQIP or NIS. Because of differences in these data sources, it may not be surprising that our results would not exactly align with the incidence information found in the literature. For instance, a 2016 study evaluated the variability in standard outcomes of posterior lumbar fusion between the University HealthSystem Consortium (UHC) and the NIS and found that the databases had similar patient populations undergoing posterior lumbar fusion, but that the UHC database reported significantly higher MI rates as well as longer lengths of hospital stay [23]. Another study by Jain et al. compared similar patient populations between a multicenter, surgeon-maintained database (SMD) and a Centers for Medicare & Medicaid Services claims database (MCD); the authors ultimately found that the incidence of post-operative AMI was slightly higher in the SMD (2.0%) than in the MCD (1.8%) [24].

Additionally, recent studies have also shown that certain variables have changed over time within both NIS and NSQIP [25–28]; thus, even comparisons within the same database can be fraught. For example, Shultz et al. reported that the 30-day incidence of post-operative MI among patients who underwent elective posterior lumbar fusion surgery with or without interbody fusion in the ACS NSQIP database changed from 0.25% over the period of 2005 to 2010 to 0.31% over the period of 2011 to 2014 [26]. Moreover, Wolf et al. conducted a retrospective review of Medicare beneficiaries who underwent primary or revisional THA between 1991 and 2008 and reported that the 90-day incidence of post-operative MI changed from 0.4% over the period of 1991 to 1993 to 0.7% over the period of 2006 to 2008; the authors speculated that this elevation was likely due to an increase in medical comorbidities among patients as well as an increase in surgical complexity [29].

### Strengths and limitations

The Optum EHR database has both inpatient and outpatient data as well as a large sample size that enabled us to generate real-world incidence estimates that are generalizable to a segment of the commercially insured US population (i.e., those in the Optum network). However, our patient population was selected to be relatively healthy, so this selection may affect the overall generalizability.

Nonetheless, this study provides additional information about AMI in a variety of risk windows. Most studies identified in the literature analyzed AMI events during index hospitalization or in the 30- or 90-day risk windows. Our study thus builds on previous work by not only estimating AMI incidences during index hospitalization and the 30- and 90-day risk windows but also by generating data on AMI incidence in the 180- and 365-day risk windows; it is important to have data in these longer risk windows to ensure no incident AMI events are missed (even though it is more likely for AMI to occur in the shorter risk windows). Lastly, our study adds to the existing literature about AMI incidence by presenting such information in the form of incidence rates; most of the AMI incidence information in the literature is presented in the form of IPs (which can be more biased due to censoring for longer follow-up periods), and thus there is a paucity of data in the form of incidence rates.

Still, it must be noted that EHR data were originally developed to improve patient care/modernize billing procedures and thus were not designed as research resources. As a result, EHR data tend to have more missing data (when compared to data obtained from clinical trials and/or prospective studies with primary data collection), and this missingness can potentially bias results [30]. However, given that elective surgery and AMI events generally require medical encounters, they would have been recorded in Optum EHR; therefore, the likelihood of missing information for these key variables would be very low. Like other studies utilizing secondary data sources without validation (e.g., medical chart review), exposure and outcome misclassification are possible. Furthermore, patients may have sought healthcare outside Optum EHR prior to the index surgery, so it is possible that a patient developed an AMI prior to the index surgery; similarly, some incident events may have been missed if a patient sought care outside the system after surgery.

Lastly, this study employed a descriptive analysis approach; thus, comparisons within stratified analyses may be subject to confounding factors that were not properly controlled. As a result, these comparisons must be interpreted with caution. Future studies in this area should consider multiple regression modeling and/or multivariable stratification techniques to better account for potential confounding. Future researchers should also consider the impact of effect measure modification on their results.

## Conclusion

This study estimated the incidence of AMI using an EHR database among adults undergoing elective SF, THA, and TKA during various post-operative risk windows and among different sub-groups. The IP of AMI following these elective procedures was generally highest among the SF cohort compared to the THA and TKA cohorts. When stratified by relevant demographic and clinical characteristics, we found that the IP of post-operative AMI was higher among patients who were older, male, with longer hospital stays, had a history of AMI, and had a history of diabetes. Future studies are warranted to confirm these findings via improved confounder control and to identify effect measure modifiers.

## Supplementary Information

**Additional file 1: Supplemental Table 1.** Incidence rate of acute myocardial infarction in patients undergoing elective spinal fusion during selected risk windows, crude and stratified by selected characteristics. **Supplemental Table 2.** Incidence rate of acute myocardial infarction in patients undergoing elective total hip arthroplasty during selected risk windows, crude and stratified by selected characteristics. **Supplemental Table 3.** Incidence rate of acute myocardial infarction in patients undergoing elective total knee arthroplasty during selected risk windows, crude and stratified by selected characteristics.

## Data Availability

The data that support the findings of this study are available from Optum but restrictions apply to the availability of these data and so are not publicly available. Data are however available from the authors upon reasonable request and with permission of Optum.
